# Comparison of physical, public and human assets as determinants of socioeconomic inequalities in contraceptive use in Colombia - moving beyond the household wealth index

**DOI:** 10.1186/1475-9276-9-10

**Published:** 2010-04-09

**Authors:** Catalina González, Tanja AJ Houweling, Michael G Marmot, Eric J Brunner

**Affiliations:** 1 Department of Epidemiology and Public Health University College London, London, UK; 2 Institute of Child Health Centre for International Health and Development, University College London, London, UK

## Abstract

**Background:**

Colombia is a lower-middle income country that faces the challenge of addressing health inequalities. This effort includes the task of developing measures of socioeconomic position (SEP) to describe and analyse disparities in health and health related outcomes. This study explores the use of a multidimensional approach to SEP, in which socioeconomic inequalities in contraceptive use are investigated along multiple dimensions of SEP. We tested the hypothesis that provision of Public capital compensated for low levels of Human capital.

**Methods:**

This study used the 2005 Colombian Demographic and Health Survey (DHS) dataset. The outcome measures were 'current non-use' and 'never use' of contraception. Inequalities in contraceptive behaviour along four measures of SEP were compared: the Household wealth index (HWI), Physical capital (housing, consumer durables), Public capital (publicly provided services) and Human capital (level of education). Principal component analysis was applied to construct the HWI, Physical capital and Public capital measures. Logistic regression models were used to estimate relative indices of inequality (RII) for each measure of SEP with both outcomes.

**Results:**

Socio-economic inequalities among rural women tended to be larger than those among urban women, for all measures of SEP and for both outcomes. In models mutually adjusted for Physical, Public and Human capital and age, Physical capital identified stronger gradients in contraceptive behaviour in urban and rural areas (Current use of contraception by Physical capital in urban areas RII 2.37 95% CI (1.99-2.83) and rural areas RII 3.70 (2.57-5.33)). The impact of women's level of education on contraceptive behaviour was relatively weak in households with high Public capital compared to households with low Public capital (Current use of contraception in rural areas, interaction p = < 0.001). Reduced educational inequalities attributable to Public capital were partly explained by differences in household wealth but not at all by health insurance cover.

**Conclusions:**

A multidimensional approach provides a framework for disentangling socioeconomic inequalities in contraceptive behaviour. We provide evidence that material circumstances indexed by Physical capital are important socioeconomic determinants while higher provision of Public capital may compensate for low levels of Human capital with respect to modern contraceptive behaviour.

## Background

Colombia is a lower-middle income country that faces the challenge of addressing health inequalities in a time of internal conflict and slow economic growth. Although Colombia fares well in regional comparisons, health inequalities in the country follow the same trends as for other countries in the region, where improvements in the average health status have been accompanied by greater relative health inequities [1-3]. Despite substantial gains in sexual and reproductive health in the country, e.g. the total fertility rate fell from 3.6 children per woman in 1986 to 2.4 in 2005, average fertility rates mask important within country inequalities in fertility rates: urban (2.3) versus rural (3.8), highest (1.5) versus lowest level of education (4.0), richest (1.7) versus poorest quintile (5.2) [4].

Disparities among regions and among people of different socioeconomic position (SEP) are particularly relevant for understanding determinants of uptake of family planning [5,6]. Effective contraception is a close determinant of fertility and as such can contribute to reducing the burden of reproductive ill health, child mortality and morbidity. Previous studies have consistently found higher contraceptive use among women with higher levels of education and other related dimensions, such as women's empowerment and autonomy, in low and middle income countries worldwide [7-12].

Addressing socioeconomic inequalities in health constitutes one of the main challenges for public health worldwide [13]. Increasing evidence of large and widening inequalities has stimulated international efforts to understand and monitor socioeconomic inequalities in various dimensions of health [14]. In low and middle income countries, these efforts include the task of developing measures of SEP in populations where data on income and expenditure have limitations in terms of availability, reliability and applicability [15-17].

Household wealth is an alternative measure of SEP widely used in low and middle income countries [17], broadly defined by asset ownership and housing quality. Wealth represents a more permanent economic status at household level than income or expenditure, because it takes into account available resources and long-run economic status [16]. The World Bank Household wealth index (HWI) of the Demographic Household Surveys (DHS) includes a broad set of assets: durable consumer goods, housing quality, water and sanitary facilities and other amenities [16]. This composite index is valuable, but captures a set of publicly provided as well as private household assets which is important to distinguish with respect to public health interventions [14]. An alternative to this limitation is a multidimensional approach, in which different dimensions of SEP are defined separately, providing a framework for attempting to disentangle causal mechanisms responsible for inequalities in health [18,19].

In research on household wealth in Latin America, asset approaches include a wider portfolio of items in comparison to literature on assets for high income countries [18-20]. In the latter, the term asset is assigned to material items with a market value, whilst in the region the term refers to tangible and intangible resources [20,21]. Similar categories of assets have been commonly grouped into domains of capital such as Human capital (e.g. level of education), Physical capital (e.g. floor materials, durable consumer goods) and Public capital (e.g. electricity, sewage) [18,22,23]. Studies in Peru, Brazil and Colombia found that access to public assets has different effects depending on women's level of education. This interaction indicates that Human capital and Public capital may complement or substitute for each other [22,24,25].

This interplay between different kinds of social inequality is a growing topic of interest in research on social inequalities in health. For example, Sen [26] proposes going beyond a unidimensional analysis where the focus is given to only one conventional measure of social stratification e.g. social class or gender, and instead study how these dimensions interact with each other. Identifying these interactions and which dimensions of socioeconomic position are stronger determinants of contraceptive use may better target effective policy interventions in family planning [25].

In this framework, the aim of this study was to examine socioeconomic inequalities in women's contraceptive use through the construction of measures that capture distinct dimensions of SEP: material circumstances (Physical capital), publicly provided assets (Public capital) and psychosocial and cognitive aspects particularly related to women's level of education (Human capital), in the DHS for Colombia of 2005. The underlying hypotheses were: a) socioeconomic inequalities in contraceptive use associated with Human capital will be larger than those by Physical capital, Public capital and the HWI, consistent with gender and health empowerment perspectives [7-12] and b) provision of Public capital compensates for low levels of Human capital (women's level of education) [22,24,25].

## Methods

### Data

The DHS are nationally-representative household surveys that provide data for a wide range of monitoring and impact evaluation indicators in the areas of population, health, and nutrition for low and middle income countries [27]. We used the Colombia 2005 DHS (version 51), which is the most recent survey conducted in the country (October 2004-June 2005). A total of 41,344 (92% response rate) women from 37,211 households were interviewed (88.4% response rate). Women of fertile age (15-49) were selected for the initial study sample (N = 38,143) (Table 1). Current-non use of contraception was estimated for women 'exposed to the risk of pregnancy' defined as fecund women (not pregnant, amenorrheic or menopausal) in union (married/cohabiting) and women not in union but sexually active in the interview month (N = 20,023) and never use of contraception was restricted to women ever sexually active (N = 32,783) (Tables S2 and S3 additional file 1). 16% of the interviewed women had missing data for the reported sexual activity and were therefore excluded from the analysis. Compared to those with recorded data, women with this missing variable were more likely to be aged 15-19 (65% vs. 10%), have achieved secondary level of education (68% vs. 48%) and be single (96% vs. 38%). The data have been described elsewhere in more detail [27,28].

**Table 1:** Distribution of women 15-49 years old for each measure of SEP by place of residence 2005 Colombian DHS

		Urban	Rural	Total
Number (%)		29337 (76.9)	8806 (23.1)	38143 (100.0)
		**% (No.)**	**% (No.)**	**% (No.)**
				
**Mean age**	Years (SD)	30.3 (10.0)	30.0 (10.1)	30.2 (10.1)
				
**Marital status**	Single	50.2 (14731)	37.8 (3325)	47.3 (18056)
	Married/cohabiting	49.8 (14606)	62.2 (5481)	52.7 (20087)
				
**Children ever born**	<2	72.2 (21193)	58.5 (5153)	69.1 (26346)
	3-4	21.3 (6247)	24.6 (2169)	22.1 (8416)
	>5	6.5 (1897)	16.9 (1484)	8.9 (3381)
				
**Household wealth (HWI)**	Richest	19.1 (5588)	1.1 (97)	14.9 (5685)
	Fourth	24.2 (7102)	3.3 (286)	19.4 (7388)
	Middle	26.9 (7882)	8.2 (726)	22.6 (8608)
	Second	23.3 (6826)	27.1 (2392)	24.2 (9218)
	Poorest	6.6 (1939)	60.2 (5305)	19.0 (7244)
				
**Physical capital**	Richest	19.7 (5768)	2.2 (189)	15.6 (5957)
	Fourth	23.7 (6954)	5.6 (492)	19.5 (7446)
	Middle	24.0 (7031)	14.2 (1250)	21.7 (8281)
	Second	20.8 (6097)	28.3 (2489)	22.5 (8586)
	Poorest	11.9 (3487)	49.9 (4386)	20.6 (7873)
				
**Public capital**	Richest	a	a	a
	Fourth	31.0 (9088)	0.6 (55)	24.0 (9143)
	Third	32.2 (9455)	8.1 (713)	26.7 (10168)
	Second	34.1 (9991)	20.6 (1816)	31.0 (11807)
	Poorest	2.7 (803)	70.7 (6222)	18.4 (7025)
				
**Human capital**	University	21.9 (6414)	4.8 (415)	17.9 (6832)
	Secondary	54.8 (16084)	37.8 (3327)	50.9 (19411)
	Primary	21.3 (6257)	50.2 (4422)	28.0 (10679)
	None	2.0 (582)	7.3 (639)	3.2 (1221)

SD: Standard deviation a. Empty quintile

### Contraceptive behaviour

The outcomes of interest in this study were 'current non-use of contraception' and 'never use of contraception'. Women were asked if they were currently using any method to delay or avoid getting pregnant at or about the time of the survey (yes/no). In addition, women were asked about their knowledge of various contraceptive methods, and those who reported knowledge of a particular method of contraception were asked if they had ever used that method (yes/no). Only modern methods of contraception were considered for the analysis (oral contraceptive, intra-uterine devices, injections, diaphragm, male/female condom, male/female sterilization, implants, foam/jelly and lactation amenorrhoea).

### A multidimensional approach to SEP

The exposures of interest were four different dimensions of SEP: the HWI, Physical capital, Public capital and Human capital (Table 2). Physical capital is measured using durable consumer goods and indicators of housing quality. Public capital is defined as access to services supplied by the state or on its behalf such as electricity and piped water. Its components capture connectedness to the public infrastructure and organisation. There are no missing values in the Colombian DHS 2005 for any of the asset variables included to construct the HWI, Physical and Public capital measures. The information on these variables was collected using the standard household questionnaire of the DHS. Respondents were asked to respond yes or no for each item listed e.g. do you have a refrigerator yes/no. The Human capital dimension is defined as women's educational attainment. Women reported their highest achieved level of education based on four categories (none/primary/secondary/university).

**Table 2:** Asset categories and components of the Household wealth index and Physical, Public and Human capital

SEP dimension	Indicator categories	Components	Household wealth index
Physical capital	Housing characteristics	Floor materials	X
		Wall materials	X
		Toilet inside/outside household	X
	
	Durable consumer goods	Shower	X
		Phone	X
		Radio	X
		TV	X
		Fridge	X
		Blender	X
		Stereo	X
		Washing machine	X
		DVD	X
		Computer	X
		Electric/gas range	X
		Electric/gas oven	X
		Microwave	X
		Vacuum or Floor polisher	X
		Hot water heater	X
		AC	X
		VCR	X
		Motorcycle or scooter	X
		Car or truck	X
		Fan	X
	
	Dwelling type	Self contained	X
		Apartment	X
		Rents in someone's home	X
		Rents in other type of building	X
		Other type	X
		No. of members per sleeping room	X
		Domestic worker	X

Public capital	Publicly provided services	Electricity	X
		Aqueduct	X
		Private toilet connected to sewer	X
		Shared toilet connected to sewer	X
		Access to natural gas	X
		Waste collected by the government	X

Human capital	Women's level of education	None	
		Primary	
		Secondary	
		University	

### Explanatory factors

Basic demographic characteristics identified in the literature [5,11] that could mediate women's contraceptive use over the course of the reproductive life cycle include urban/rural place of residence, women's age in years, health insurance (no/yes), marital status categorised into two groups (single/married-cohabiting) and number of children ever born categorised into three groups (<2/3-4/>5) (Table 1).

### Construction of the measures of SEP

Each measure of SEP was constructed by applying the following steps: selection of the indicators (Table 2), coding of variables, calculation of weights, construction of an index using these weights, and classification of households into SEP groups (quintiles). In the case of Human capital with only one component variable, only the first two steps apply.

The weights used to construct the indices were derived through principal component analysis (PCA) using Filmer and Pritchett's approach [29]. Most of the assets variables collected in the Colombian DHS 2005 were categorical variables. To include them in the PCA qualitative categorical variables were re-coded into binary variables (no/yes). To examine the distribution for each index, a histogram with kernel-density estimates was generated (Figure S1, Figure S2, Figure S3 additional file 1). Sample weights were not used for the PCA, but were used when constructing SEP quintiles for the HWI, Physical capital and Public capital measures. The stability of the categorization into quintiles was assessed by comparing household classification by Physical assets and Public assets against the HWI. The percentage of households that remained in the same quintile and those that moved one, two or three quintiles were calculated through cross-tabulation. Additionally, spearman rank correlations between the HWI and Physical and Public capital quintiles were computed.

To assess the internal coherence of the HWI, Physical capital and Public capital measures, the mean value for each specific item was compared between quintiles. Internal coherence was defined as the agreement of the distribution of assets and services across quintiles [17]. For assessing reliability, the household sample was split into random halves to run the PCA of each asset based measure on each and factor loadings were visually compared (data not shown here can be found in [30]).

### Data analysis

Inequalities in non-use of modern contraceptives were measured using the Relative Index of Inequality (RII) [31]. The RII is a commonly used measure in the study of social inequalities in health, which takes into account the size and relative position of the wealth groups. For the construction of RII the categories of each measure of SEP were hierarchically organized from the richest to the poorest quintile and in the case of the Human capital, from the highest level of education to no education level. Each measure of SEP is converted to a continuous distribution between 0 (highest SEP) and 1(lowest SEP). The distribution is weighted according to the population in each SEP group by calculating the midpoint of the proportion in each category e.g. if 10% of the sample were in the highest social group and 15% were in the next category, those in the highest would be given a value of 0.05 (0.10/2) and those in the next group would receive a value of 0.175 (0.10+0.15/2). The Relative Index of Inequality (RII) is obtained by regressing each measure of SEP on a binary outcome in a logistic regression model [31]. A large score on the RII implies large socioeconomic inequalities for the outcome under study. Likelihood ratio tests were used to test the difference in RII by place of residence (urban/rural).

Crude and adjusted RII with 95% confidence intervals were estimated. The simplest model was adjusted for age by entering age in years as a continuous variable, marital status (single/married-cohabiting) and number of children ever born (<2/3-4/>5); subsequently it was stratified by place of residence (urban/rural). The final model was mutually adjusted for Physical capital, Public capital, and Human capital to estimate the independent contributions of each measure of SEP. The HWI was not included in this model in view of collinearity, as the Physical and Public capital items are contained in the HWI.

Likelihood ratio tests were used to test for interactions between Human capital, the weighted distribution of women's level of education and Public capital (Low/High) separately for urban and rural women and adjusted for age in years as a continuous variable, marital status (single/married-cohabiting), and number of children ever born (<2/3-4/>5). To show the effect of each level of education odds ratios were calculated using three groups (University-Secondary, Primary and None) due to insufficient data especially in rural areas where there is a lower proportion of women with higher levels of education. Public capital quartiles were divided into two halves (low/high). We then added Physical capital and health insurance (no/yes) and assessed their effect in the interaction model and tested for trends. Health insurance had no effect and was omitted from the model. We calculated the percent change in the coefficients for the effect of University/High compared to no education in models with and without adjustment for physical capital. All statistical analyses were conducted using Stata version 10.0 (Stata Inc., TX, USA).

## Results

77% of the respondents lived in urban areas and 23% in rural areas (Table 1). There were large rural-urban inequalities in Physical, Human and Public capital, with rural women being poorer, less educated, and with less access to Public capital compared to urban women. In addition, inequalities in these forms of capital were larger within rural areas than within urban areas (Table 1).

The extent to which households could be distributed into population quintiles differed between the measures of SEP. It was possible to differentiate five groups for the HWI and Physical capital, and four groups for Public capital (Table 1). Compared to HWI quintiles, agreement was strongest for Physical capital quintiles (Spearman rank correlation 0.92) with 75% in the same quintile, 24% moving one quintile and 1% moving two or three quintile groups. Agreement was weaker with Public capital quintiles (Spearman rank correlation 0.72) with 46% in the same quintile, 35% moving one quintile and 19% moving two or three quintiles.

There was evidence of internal coherence for all measures of SEP when comparing the mean value for each asset variable by quintiles. For example, for the HWI 41% of the poorest households, 82% in the middle households and, 90% of the richest households had access to piped water. In the case of durable assets, a refrigerator was owned by 17% of the poorest households, 78% of the middle households and, 99% of the richest households. The proportion of households with dirt walls, earth/mud floors, and connected to septic systems decreased for each richer quintile. Spearman rank correlations between the Physical, Public and Human measures ranged from 0.08 to 0.41 in urban areas and 0.19 to 0.32 in rural areas. When the sample was split into random halves, the PCA loadings and direction of the loadings were similar in each half for each asset-based measure of SEP.

### Socioeconomic inequalities in contraceptive use

Table 3 shows that the adjusted prevalence of current non-use and never use of contraception was respectively higher for women in rural areas (28%, 17%) compared to those in urban areas (24%, 9%) (difference p-values <0.001). Reported non-use generally decreased from the poorest to the richest for each measure of SEP with some small deviations, e.g. current non-use by HWI among women in the middle quintile (18%) compared to women in the fourth richer quintile (24%) in rural areas.

**Table 3:** Adjusted prevalence of modern contraceptive use and RII (95% CI) by each measure of SEP

		Prevalence (%)		RII (95% CI)		Difference
		Urban	Rural	Urban	Rural	p-value
**Current non-use of contraception among women in ** **union (married/cohabiting) and single sexually active**		23.7	28.2	**N = 15147**	**N = 4876**	<0.001
Household wealth (HWI)	Richest	19.8	13.7			
	Fourth	21.0	23.7			
	Middle	23.7	17.5	2.84 (2.41-3.35)	4.6 (3.06-6.79)	<0.001
	Second	28.0	23.4			
	Poorest	30.4	33.5			
						
Physical capital	Richest	21.0	16.9			
	Fourth	21.1	20.3			
	Middle	22.5	19.6	2.64 (2.26-3.07)	3.96 (2.82-5.55)	<0.001
	Second	28.0	26.7			
	Poorest	29.6	33.9			
						
Public capital	Richest	a	a			
	Fourth	22.3	17.7			
	Third	22.3	21.2	1.84 (1.56-2.17)	2.01 (1.35-2.97)	0.09
	Second	26.2	26.9			
	Poorest	30.8	29.7			
						
Human capital	University	23.0	20.8			
	Secondary	22.9	24.1			
	Primary	25.3	28.6	1.44 (1.24-1.67)	1.64 (1.14-2.37)	<0.001
	None	25.2	51.4			
						
**Never use of contraception among ever-sexually active women**		8.9	17.2	**N = 25231**	**N = 7552**	<0.001
Household wealth (HWI)	Richest	4.9	3.8			
	Fourth	7.3	7.2			
	Middle	9.0	8.0	7.14 (5.94-8.59)	23.5 [14.6-38.1]	<0.001
	Second	10.9	12.5			
	Poorest	18.5	22.8			
						
Physical capital	Richest	5.3	5.3			
	Fourth	7.4	6.9			
	Middle	8.6	9.0	5.81 (4.89-6.91)	19.63 [13.13-29.35]	<0.001
	Second	10.6	14.5			
	Poorest	15.2	24.1			
						
Public capital	Richest	a	a			
	Fourth	8.3	7.3			
	Third	7.3	9.1	2.47 (2.04-2.99)	7.72 [4.93-12.10]	<0.001
	Second	10.6	12.3			
	Poorest	15.4	20.2			
						
Human capital	University	6.3	9.1			
	Secondary	8.3	12.0			
	Primary	12.3	19.7	4.83 (4.04-5.78)	7.98 (5.27-12.10)	<0.001
	None	22.3	43.8			

a. Empty quintile []. Large confidence intervals possibly due to uneven distribution of women between quintile groups see Table S3 additional file 1.

Note: Prevalence and RII adjusted for age in years, marital status and number of children ever born by place of residence.

Urban and rural areas have different levels of inequality in contraceptive behaviour. There was evidence of inequalities for both outcomes with all four measures of SEP among women in urban and rural areas. Inequalities were significantly larger for women living in rural areas compared to women in urban areas for each measure of SEP (difference p-values < 0.001), except by Public capital for current non-use of contraception (Table 3). In urban areas, inequalities were wider by HWI for current non-use of contraception (RII 2.84 95% CI 2.41-3.35) and never use of contraception (RII 7.14 95% CI 5.94-8.59). In rural areas, there were large inequalities in contraceptive behaviour by Physical, Public and Human capital but inequalities were wider by HWI.

A model mutually adjusted for Physical, Public and Human capital and age in years, marital status and children ever born (Table 4) suggests that Physical capital tended to be the stronger socioeconomic determinant of contraceptive behaviour in urban and rural areas. Public and Human capital showed substantial and statistically significant inequalities for never use of contraception among women in rural areas.

**Table 4:** RII (95% CI) for current non-use and never use of modern contraceptive methods

	RII (95% CI)			p-value
		
	Urban		Rural	
**Current non-use among women in union (married/cohabiting) and single sexually active**				
	**(N = 15147)**		**(N = 4876)**	
Physical capital	2.37 (1.99-2.83)	<0.001	3.70 (2.57-5.33)	<0.001
Public capital	1.28 (1.07-1.53)	0.01	1.25 (0.82-1.89)	0.30
Human capital	1.05 (0.90-1.24)	0.51	1.04 (0.71-1.54)	0.83
**Never use of contraception among ever-sexually active women**				
	**(N = 25231)**		**(N = 7552)**	
Physical capital	3.70 (3.02-4.52)	<0.001	11.23 [7.36-17.16]	<0.001
Public capital	1.19 (0.96-1.48)	0.10	2.83 (1.76-4.54)	<0.001
Human capital	3.02 (2.50-3.65)	<0.001	3.41 (2.24-5.21)	<0.001

[]. Large confidence intervals possibly due to uneven distribution of women between quintile groups see Table S3 additional file 1.

Note: Mutually adjusted for Physical, Public, Human capital, age in years, marital status and number of children ever born by place of residence.

Table 5 shows results for the hypothesis that provision of Public capital compensates for low levels of Human capital. There was a strong association between education and contraceptive use in households with low and high levels of Public capital, such that women with lower education reported higher non-use of contraception.

**Table 5:** Effect of women's level of education on modern contraceptive use in households with low and high provision of Public capital

		OR (95% CI) p-value					
		
Level of Public capital	Level of education		Urban			Rural	
			(N = 15147)			(N = 4876)	
		**Current non-use of contraception among women in union (married/cohabiting) and single sexually active**					
		N			N		
**Low Public capital**	**University/Secondary**	3806	1		1497	1	
	**Primary**	1541	1.18 (1.02-1.36)	0.03	2607	1.09 (0.93-1.28)	0.28
	**None**	160	1.66 (1.14-2.42)	0.01	338	2.53 (1.93-3.32)	<0.001
							
	*Trend*			0.17			<0.001
							
**High Public capital**	**University/Secondary**	7423	1		243	1	
	**Primary**	2092	1.06 (0.94-1.21)	0.36	183	0.84 (0.52-1.37)	0.48
	**None**	125	1.10 (0.68-1.76)	0.71	8	1.16 (0.22-6.01)	0.86
							
	*Trend*			0.16			0.54
	**Interaction**			0.91			<0.001
			**(N = 25231)**			**(N = 7552)**	
		**Never use of contraception among ever sexually active women**					
		N			N		
**Low Public capital**	**University/Secondary**	6464	1		2451	1	
	**Primary**	2587	1.81 (1.55-2.13)	<0.001	3841	1.51 (1.28-1.77)	<0.001
	**None**	330	4.14 (2.98-5.77)	<0.001	582	5.82 (4.55-7.44)	<0.001
							
	*Trend*			<0.001			<0.001
							
**High Public capital**	**University/Secondary**	12247	1		393	1	
	**Primary**	3376	1.66 (1.44-1.92)	<0.001	269	1.01 (0.56-1.79)	0.99
	**None**	227	2.13 (1.34-3.89)	0.001	16	1.84 (0.39-8.63)	0.44
							
	*Trend*			<0.001			0.46
	**Interaction**			0.12			0.10

Note: Adjusted for age in years, marital status, number of children ever born and Physical capital by place of residence. Trend and interaction tests based on weighted Human capital (level of education) distribution.

There was evidence of interaction between Human capital (level of education) and Public capital (Low/High) for current non-use of contraception in women living in rural areas in a model adjusted for age in years, marital status and children ever born. These interaction effect remained after adjusting for Physical capital (household wealth) (Table 5). Households with higher Public capital were wealthier in terms of Physical capital in urban and rural areas (urban areas: 0.79 SD wealthier on physical capital score; rural areas: 1.03 SD; unpaired t test p < 0.001 for both areas) Adjustment for Physical capital attenuated inequalities in current non-use of contraception according to level of education (15-62%). When self-reported health insurance cover was controlled for in the interaction model, unadjusted for Physical capital, the coefficients did not change. Results from sensitivity tests on all women of fertile age (15-49) showed a similar gradient (data not shown).

## Discussion

### Main findings

A multidimensional approach [18,32], in which different dimensions of SEP are measured separately, provides a framework for disentangling socioeconomic inequalities in health in a way that is not possible with a composite index such as the widely used World Bank HWI. In Colombia, we show that inequalities in contraceptive behaviour by Physical capital were larger than by Public and Human capital dimensions of SEP. Inequalities in never use of modern contraceptive methods associated with Public and Human capital were also important, especially for women in rural areas. Importantly, the impact of education on contraceptive behaviour tended to be weaker for households with high access to Public capital and stronger in households with low access to Public capital. As far as the authors know, this is the first time this approach is used to analyse Colombian data. In low and middle income countries where monitoring poor-rich inequalities in health has become a central policy objective, for example in the context of the Millennium development Goals [33], a multidimensional approach provides an alternative theory-driven use of existing survey asset data that moves beyond a one-dimensional measure of SEP to provide understanding into the effects of multiple dimensions of SEP in health inequalities.

### Multidimensional framework

To construct the asset-based measures of SEP the composite HWI was divided into two dimensions: material (Physical capital) and publicly provided services (Public capital), and a third dimension was added based on the level of women's educational attainment (Human capital). Previous studies have used similar dimensions to construct asset-based measures of SEP in the absence of data on income and/or expenditure [18,22,32]. We found that for contraceptive behaviour, the magnitude of inequalities varied with each of the dimensions of SEP studied. Houweling et al. [14] explored the HWI and three alternative asset-based indices as measures of inequality in under-5 mortality and measles immunisation. They found that the observed poor-rich differences in both outcomes were sensitive to the measure used. The size and direction of change varied per country, index and health outcome. Others have observed similar results [14,34].

We have found that grouping a large number of assets into coherent dimensions compared to the composite HWI, facilitates an intermediate level of analysis [18], by comparing the magnitude of inequalities in contraceptive behaviour for each dimension. In Colombia, our findings illustrate that for women in rural areas the magnitude of inequalities in contraceptive behaviour were larger in all SEP dimensions when compared to urban women. This approach is attractive because it can allow a different set of questions to be asked; instead of focusing exclusively on economic social class or women's level of education as determinants of contraceptive behaviour we can analyse the relative importance of different types of socioeconomic inequalities (e.g. Human, Physical and Public capital) and how these may vary in different places of residence [26,32].

For the Public capital measure, there is clumping and truncation in the distribution, explained by the few indicators available. This gap makes it impossible to distinguish between the households in urban and rural samples that report access to all services (see Table S1 additional file 1) [17,32]. Houweling et al [14] found a similar result and explained this phenomenon on the basis of the choice of variables included in the index. Our results suggest that additional indicators of public services e.g. access to public transport and road infrastructure at the household and community level should be considered to refine the stratification amongst the richer groups, both in urban and rural areas [23].

Women's level of education was selected as the only component of Human capital because information on education, either measured as years of education or achieved level of education, is the most widely used proxy for Human capital in Latin America [18,20,22]. Other possible indicators of Human capital include information on partner's level of education and occupation of household members. Our approach used Human capital as an individual level measure, whereas HWI, Physical and Public SEP dimensions correspond to household level information. The use of a household level measure of Human capital is a possible future direction in a multilevel framework [35].

### Explaining socioeconomic inequalities in modern contraceptive use

This study shows that a multidimensional asset-based approach provides a theoretical advantage in health inequalities research. Separating different dimensions of SEP and studying their interaction effects enhance the extent to which we are able to explain these inequalities. This study found that measures of contraceptive use were strongly associated with SEP dimensions that reflect material pathways (Physical capital, Public capital) and psychosocial pathways (Human capital) as well as the composite HWI. These findings suggest that the well known effects of women's education on contraceptive behaviour [9] were confirmed in this study, particularly for women's lifetime prevalence of modern contraceptive use. The literature on the education-fertility relationship has consistently shown that the experience of education has a lasting impact for women's lives that serves as a resource of knowledge and empowerment, as a vehicle of socioeconomic mobility and as a modifier of attitudes that influence women's reproductive desires and behaviour [5,7-10]. Importantly, in rural areas all three dimensions of SEP, Human, Public and Physical capital identified inequalities in contraceptive use. This finding suggests that in addition to education, material living conditions and access to publicly provided services play an important role in women's contraceptive behaviour, particularly for current non-use of modern contraceptive methods. These results do not undermine the importance of education, but point out to the cyclical relationship between disadvantaged living conditions, lower educational levels, and higher fertility trends observed in the region; mainly in rural areas and in urban slums with severe lack of public infrastructure [6,11].

Besides asking which socioeconomic dimensions are important for women with respect to their contraceptive behaviour, we also investigated how these dimensions may interact and for which social groups. Our findings of an interaction between Human capital and Public capital in rural areas suggests that provision of public services to the household has a compensatory effect for women with lower levels of Human capital with respect to contraceptive behaviour. Similarly, a study in Peru found an interaction between Public service availability at household level and maternal years of education with respect to their children's nutritional status. Nutritional status was higher among children in households with access to public services compared to those without them when mothers had less years of education, but this contrast was not evident among more educated mothers [22]. These important observations emphasize that public provision of infrastructure could substitute or complement the effect of level of education among women who lacked educational opportunity in relation to many aspects of personal and family health [22,24,25].

The interaction between level of education and Public capital in relation to contraceptive behaviour in DHS 2005 takes the expected form. Among households with high Public capital the education gradient is smaller, while among households with low Public capital it is larger. There may be two explanations for our findings. First, women in households with high Public capital may have better access to family planning through health insurance [11], yet the inclusion of health insurance cover had no effect in the interaction model. Second, better living conditions may influence contraceptive use through higher physical wealth and resources in the household [36]. Households with high Public capital do differ from low Public capital households particularly in terms of physical wealth (ownership of durable goods and housing quality) in urban and rural areas. However, we demonstrate that the interaction remains after adjusting for household wealth (Physical capital) consistent with an independent effect of public services provision.

On the other hand, the combined effects of low levels of education (Human capital) and low Public capital may operate as a bottleneck for family planning interventions in deprived urban and rural areas in Colombia. The evidence that higher provision of Public capital compensates for low levels of Human capital suggests that government investment in public services is even more necessary in areas where women with lower levels of education are clustered [22]. The socioeconomic gap in contraceptive use documented in Colombia in the past decade [6] is likely to decrease with improvement of household living conditions and community infrastructure. Other dimensions such as availability and accessibility to family planning programmes, domestic violence, ethnicity, religious attitudes and cultural norms about sexual and reproductive health, could be key to understanding our findings, especially in rural areas in Colombia where the Catholic Church remains a strong influence on family planning [12,37].

Future studies could investigate two other possible mechanisms. First, Public capital could be a proxy for local economic development, with better public infrastructure and social organisation such as health programmes or services in those areas that have mains water, sewage and electricity [35]. Households with higher Public capital may be more exposed to family planning campaigns, closer to pharmacies and hospitals and other factors associated to family planning uptake [6,12]. Second, higher levels of Public capital could benefit women in their household chores (e.g. household access to water, garbage collection) and indirectly provide women with autonomy that may translate into spare time to participate in activities that enhance women's health and status e.g. social activities and use of health services [36].

### Limitations

The results of this study should be carefully interpreted for the Colombian population as some marginalized groups, such as the internally displaced population, are likely to be underrepresented in the sample. Another limitation is the restriction on asset data for the construction of the asset-based measures of SEP. The DHS for Colombia 2005 was not designed for collecting information on assets, and although the indicators included cover a wide range of assets, information on other type of assets, such as livestock and land ownership, relevant for assessing SEP are absent from the data [14,15,17]. Secondly, health insurance coverage is a poor proxy for access to family planning as the most common sources of contraceptive methods in Colombia are pharmacies and Profamilia, a private family planning agency [6]. Finally, the definition of 'being at risk of pregnancy' for single women on the basis of women's report of recent sexual activity is debatable i.e. highly educated single women might be less likely to report sexual activity. The exclusion of single women with a non-response to sexual activity in the month of interview could lead to biased results.

## Conclusions

A multidimensional asset-based approach provides a framework for disentangling socioeconomic inequalities in contraceptive behaviour. Its application facilitates an intermediate level of analysis between a composite index (HWI) and multiple measures of SEP, by comparing the magnitude of health inequalities attributable to specific dimensions. We believe this approach could be a starting point to address questions about the relative importance of different dimensions of SEP on inequalities in health. For women living in urban and rural areas in Colombia we have shown that Physical capital identified important socioeconomic inequalities in current non-use and never use of modern contraceptive methods. Importantly, we provide some support for our interaction hypothesis that provision of public services compensates for women's low levels of education with respect to contraceptive behaviour. Our results suggest that women's education and household living conditions should be continued and strengthen in public health policy objectives for Colombia. If complemented with wider provision of public infrastructure in deprived urban and rural areas of the country, socioeconomic inequalities in modern contraceptive use may be reduced.

## Competing interests

The authors declare that they have no competing interests.

## Authors' contributions

CG and EB jointly developed the principal research idea for the analyses. CG obtained and analysed the data and reviewed the literature. CG and TH drafted the paper. EB supervised the analyses, and all authors read and approved the final manuscript.

## Supplementary Material

Additional file 1 Table S1, Table S2, Table S3 and Figure S1, Figure S2 and Figure S.3. Click here for file
