# Supplementary material for: Comparison of physical, public and human assets as determinants of socioeconomic inequalities in contraceptive use in Colombia - moving beyond the household wealth index
Source: Int J Equity Health. 2010 Apr 9;9:10. doi: 10.1186/1475-9276-9-10 (PMC2873465; doi:10.1186/1475-9276-9-10)
Supplement: Additional file 1 — Table S1, Table S2, Table S3 and Figure S1, Figure S2 and Figure S.3. [file 1475-9276-9-10-S1.DOC]

**Table S1 Prevalence (%) of components of the Household Wealth Index in all urban and rural households Colombia DHS 2005**

|  | **Urban**  **N=27794** | | | | |  | **Rural**  **N=9417** | | | | |
| --- | --- | --- | --- | --- | --- | --- | --- | --- | --- | --- | --- |
|  | **Poorest** | **Second** | **Middle** | **Fourth** | **Richest** |  | **Poorest** | **Second** | **Middle** | **Fourth** | **Richest** |
| **Source of drinking water** |  |  |  |  |  |  |  |  |  |  |  |
| Aqueduct | 41 | 70 | 82 | 88 | 90 |  | 11 | 34 | 55 | 71 | 79 |
| Public stand pipe | 1 | 0 | 0 | 0 | 0 |  | 0 | 0 | 0 | 0 | 0 |
| Well with pump | 10 | 9 | 5 | 2 | 1 |  | 3 | 4 | 4 | 2 | 1 |
| Well without pump | 3 | 1 | 0 | 0 | 0 |  | 12 | 2 | 1 | 0 | 0 |
| River, stream, pond, lake | 3 | 0 | 0 | 0 | 0 |  | 20 | 5 | 1 | 0 | 0 |
| Tanker truck | 0 | 0 | 0 | 0 | 0 |  | 0 | 1 | 1 | 0 | 0 |
| Cart with small tank | 2 | 1 | 1 | 0 | 0 |  | 1 | 1 | 2 | 2 | 0 |
| Bottled | 4 | 9 | 9 | 8 | 8 |  | 1 | 3 | 6 | 9 | 15 |
| Rainwater | 29 | 7 | 2 | 1 | 0 |  | 10 | 4 | 3 | 3 | 1 |
| **Toilet type** |  |  |  |  |  |  |  |  |  |  |  |
| Connected to sewer | 14 | 39 | 73 | 92 | 97 |  | 5 | 36 | 72 | 82 | 82 |
| Shared connected to sewer | 12 | 32 | 17 | 5 | 1 |  | 2 | 9 | 6 | 3 | 2 |
| Connected to septic system | 30 | 18 | 8 | 3 | 1 |  | 35 | 38 | 18 | 13 | 15 |
| Shared connected septic system | 7 | 4 | 1 | 0 | 0 |  | 4 | 3 | 0 | 0 | 1 |
| Unconnected flush toilet | 4 | 2 | 1 | 0 | 0 |  | 6 | 5 | 2 | 2 | 0 |
| Shared unconnected flush toilet | 2 | 1 | 0 | 0 | 0 |  | 1 | 0 | 0 | 0 | 0 |
| Pit latrine | 4 | 1 | 0 | 0 | 0 |  | 6 | 1 | 0 | 0 | 0 |
| Shared pit latrine | 1 | 0 | 0 | 0 | 0 |  | 1 | 0 | 0 | 0 | 0 |
| Pipe to sea | 2 | 1 | 1 | 0 | 0 |  | 2 | 3 | 1 | 0 | 0 |
| Shared pipe to sea | 1 | 0 | 0 | 0 | 0 |  | 0 | 0 | 0 | 0 | 0 |
| Bush | 23 | 2 | 0 | 0 | 0 |  | 39 | 2 | 0 | 0 | 0 |
| **Durable consumer goods** |  |  |  |  |  |  |  |  |  |  |  |
| Shower | 13 | 60 | 87 | 96 | 99 |  | 73 | 74 | 91 | 95 | 98 |
| Phone | 4 | 21 | 54 | 86 | 97 |  | 5 | 23 | 45 | 70 | 90 |
| Radio | 38 | 52 | 68 | 80 | 89 |  | 57 | 66 | 75 | 83 | 90 |
| TV | 47 | 73 | 95 | 99 | 100 |  | 48 | 87 | 98 | 99 | 100 |
| Refrigerator | 17 | 40 | 78 | 96 | 99 |  | 21 | 65 | 91 | 97 | 100 |
| Blender | 23 | 46 | 79 | 96 | 99 |  | 30 | 69 | 90 | 98 | 100 |
| Stereo | 6 | 15 | 35 | 65 | 90 |  | 8 | 31 | 57 | 80 | 95 |
| Washing machine | 1 | 4 | 11 | 32 | 81 |  | 1 | 7 | 17 | 43 | 80 |
| Digital Versatile Disc (DVD) player | 1 | 2 | 6 | 13 | 44 |  | 1 | 4 | 13 | 21 | 60 |
| Computer | 0 | 1 | 2 | 9 | 48 |  | 0 | 1 | 4 | 11 | 59 |
| Internet | 0 | 0 | 1 | 2 | 24 |  | 0 | 1 | 1 | 2 | 31 |
| Cooking range | 43 | 83 | 96 | 98 | 99 |  | 38 | 90 | 98 | 99 | 99 |
| Vacuum/buffer | 0 | 0 | 0 | 2 | 22 |  | 0 | 0 | 1 | 3 | 36 |
| Microwave | 0 | 0 | 1 | 4 | 31 |  | 0 | 1 | 3 | 10 | 55 |
| Hot water heater | 0 | 0 | 2 | 7 | 32 |  | 0 | 2 | 8 | 9 | 51 |
| Air condition | 0 | 0 | 0 | 1 | 10 |  | 0 | 0 | 1 | 3 | 8 |
| Video Cassette Recorder | 0 | 1 | 3 | 6 | 37 |  | 0 | 2 | 4 | 15 | 45 |
| Motorcycle/Scooter | 3 | 6 | 12 | 18 | 25 |  | 2 | 9 | 15 | 24 | 31 |
| Car/Truck | 0 | 1 | 3 | 7 | 36 |  | 1 | 5 | 9 | 20 | 53 |
| Fan | 37 | 48 | 50 | 46 | 49` |  | 20 | 31 | 31 | 41 | 44 |
| **Dwelling type** |  |  |  |  |  |  |  |  |  |  |  |
| Separate house | 91 | 79 | 79 | 78 | 70 |  | 98 | 94 | 93 | 90 | 85 |
| Apartment | 1 | 6 | 16 | 21 | 30 |  | 1 | 2 | 5 | 10 | 15 |
| Rents in someone’s home | 5 | 11 | 3 | 0 | 0 |  | 1 | 3 | 1 | 0 | 0 |
| Rents in other type | 2 | 4 | 2 | 1 | 0 |  | 1 | 1 | 0 | 0 | 0 |
| **Floor material** |  |  |  |  |  |  |  |  |  |  |  |
| Parquet/polished wood | 0 | 0 | 1 | 1 | 3 |  | 0 | 0 | 0 | 1 | 3 |
| Carpet | 0 | 0 | 0 | 0 | 2 |  | 0 | 0 | 0 | 0 | 2 |
| Tiles | 0 | 12 | 34 | 73 | 89 |  | 3 | 17 | 40 | 76 | 82 |
| Wood planks | 21 | 8 | 4 | 2 | 2 |  | 15 | 8 | 4 | 1 | 2 |
| Cement | 46 | 75 | 60 | 23 | 4 |  | 47 | 72 | 55 | 23 | 10 |
| Earth/mud/dung/sand | 32 | 5 | 1 | 0 | 0 |  | 35 | 3 | 1 | 0 | 0 |
| **Wall material** |  |  |  |  |  |  |  |  |  |  |  |
| Adobe | 4 | 3 | 2 | 1 | 0 |  | 8 | 6 | 5 | 2 | 1 |
| Bahareque | 10 | 5 | 2 | 1 | 0 |  | 21 | 8 | 3 | 0 | 0 |
| Rough wood walls | 45 | 10 | 1 | 0 | 0 |  | 24 | 4 | 1 | 0 | 0 |
| Cane walls | 3 | 1 | 0 | 0 | 0 |  | 5 | 1 | 0 | 0 | 0 |
| Brick/block/wood | 31 | 78 | 94 | 98 | 99 |  | 36 | 79 | 90 | 97 | 99 |
| Dirt | 2 | 2 | 1 | 0 | 0 |  | 5 | 3 | 1 | 0 | 0 |
| Flimsy | 5 | 1 | 0 | 0 | 0 |  | 1 | 0 | 0 | 0 | 0 |
| **Waste management** |  |  |  |  |  |  |  |  |  |  |  |
| Collected by government | 51 | 90 | 96 | 98 | 99 |  | 5 | 47 | 87 | 95 | 98 |
| Burned | 27 | 4 | 0 | 0 | 0 |  | 49 | 30 | 7 | 4 | 0 |
| Buried | 3 | 1 | 0 | 0 | 0 |  | 6 | 4 | 1 | 0 | 0 |
| Dumped in waterways | 6 | 1 | 0 | 0 | 0 |  | 8 | 2 | 0 | 0 | 0 |
| Dumped in compound/yard | 10 | 1 | 0 | 0 | 0 |  | 29 | 11 | 1 | 1 | 1 |
| Informally | 2 | 4 | 3 | 2 | 1 |  | 1 | 5 | 3 | 1 | 1 |

## Table S2 –Distribution of fecund women in union (married/cohabiting) and single sexually active for each measure of SEP by place of residence 2005 Colombian DHS

|  |  | **Urban** | **Rural** | **Total** |
| --- | --- | --- | --- | --- |
| **Number (%)** |  | **15147 (75.7)** | **4876 (24.4)** | **20023 (100.0)** |
|  |  |  |  |  |
|  |  | **% (No.)** | **% (No.)** | **% (No.)** |
|  |  |  |  |  |
| **Mean age** | Years (SD) | 32.4 (8.9) | 32.8 (8.9) | 32.5 (8.9) |
|  |  |  |  |  |
| **Marital status** | Single | 21.5 (3255) | 10.2 (499) | 18.8 (3754) |
|  | Married/cohabiting | 78.5 (11892) | 89.8 (4377) | 81.3 (16269 |
|  |  |  |  |  |
| **Children ever born** | <2 | 63.4 (9610) | 44.8 (2185) | 58.9 (11795) |
|  | 3-4 | 28.6 (4328) | 33.4 (1630) | 29.8 (5958) |
|  | >5 | 8.0 (1209) | 21.8 (1061) | 11.3 (2270) |
|  |  |  |  |  |
| **Household wealth (HWI)** | Richest | 19.5 (2955) | 1.2 (60) | 15.1 (3015) |
|  | Fourth | 24.6 (3730) | 3.5 (169) | 19.5 (3899) |
|  | Middle | 26.8 (4060) | 8.2 (402) | 22.3 (4462) |
|  | Second | 23.4 (3537) | 28.3 (1380) | 24.6 (4917) |
|  | Poorest | 5.7 (865) | 58.8 (2865) | 18.6 (3730) |
|  |  |  |  |  |
| **Physical capital** | Richest | 20.2 (3062) | 2.5 (120) | 15.9 (3182) |
|  | Fourth | 24.1 (3643) | 5.8 (283) | 19.6 (3926) |
|  | Middle | 23.7 (3585) | 14.7 (716) | 21.5 (4301) |
|  | Second | 21.2 (3206) | 29.3 (1429) | 23.2 (4635) |
|  | Poorest | 10.9 (1651) | 47.7 (2328) | 19.9 (3979) |
|  |  |  |  |  |
| **Public capital** | Richest | a | a | a |
|  | Fourth | 30.4 (4597) | 0.6 (28) | 23.1 (4625) |
|  | Third | 33.3 (5043) | 8.3 (406) | 27.2 (5449) |
|  | Second | 33.7 (5108) | 20.6 (1002) | 30.5 (6110) |
|  | Poorest | 2.6 (399) | 70.6 (3440) | 19.2 (3839) |
|  |  |  |  |  |
| **Human capital** | University | 22.0 (3327) | 4.2 (204) | 17.6 (3531) |
|  | Secondary | 52.2 (7902) | 31.5 (1536) | 47.1 (9438) |
|  | Primary | 24.0 (3633) | 57.2 (2790) | 32.1 (6423) |
|  | None | 1.9 (285) | 7.1 (346) | 3.2 (631) |

SD: Standard deviation a. Empty quintile

## Table S3 –Distribution of ever sexually active women for each measure of SEP by place of residence 2005 Colombian DHS

|  |  | **Urban** | **Rural** | **Total** |
| --- | --- | --- | --- | --- |
| **Number (%)** |  | **25231 (77.0)** | **7552 (23.0)** | **32783 (100.0)** |
|  |  |  |  |  |
|  |  | **% (No.)** | **% (No.)** | **% (No.)** |
|  |  |  |  |  |
| **Mean age** | Years (SD) | 32.0 (9.4) | 31.9 (9.4) | 32.0 (9.4) |
|  |  |  |  |  |
| **Marital status** | Single | 42.1 (10625) | 27.4 (2071) | 38.7 (12696) |
|  | Married/cohabiting | 57.9 (14606) | 72.6 (5481) | 61.3 (20087) |
|  |  |  |  |  |
| **Children ever born** | <2 | 67.7 (17087) | 51.6 (3899) | 64.0 (20986) |
|  | 3-4 | 24.8 (6247) | 28.7 (2169) | 25.7 (8416) |
|  | >5 | 7.5 (1897) | 19.7 (1484) | 10.3 (3381) |
|  |  |  |  |  |
| **Household wealth (HWI)** | Richest | 18.7 (4717) | 1.2 (89) | 14.7 (4806) |
|  | Fourth | 23.9 (6032) | 3.2 (242) | 19.1 (6274) |
|  | Middle | 27.0 (6806) | 8.3 (627) | 22.7 (7433) |
|  | Second | 23.7 (5978) | 27.0 (2041) | 24.5 (8019) |
|  | Poorest | 6.7 (1698) | 60.3 (4553) | 19.1 (6251) |
|  |  |  |  |  |
| **Physical capital** | Richest | 19.3 (4878) | 2.2 (164) | 15.4 (5042) |
|  | Fourth | 23.5 (5931) | 5.6 (422) | 19.4 (6353) |
|  | Middle | 23.9 (6038) | 14.0 (1054) | 21.6 (7092) |
|  | Second | 21.0 (5303) | 28.1 (2124) | 22.7 (7427) |
|  | Poorest | 12.2 (3081) | 50.2 (3788) | 21.0 (6869) |
|  |  |  |  |  |
| **Public capital** | Richest | a | a | a |
|  | Fourth | 30.0 (7573) | 0.7 (52) | 23.3 (7625) |
|  | Third | 32.8 (8277) | 8.3 (626) | 27.2 (8903) |
|  | Second | 34.4 (8686) | 20.5 (1547) | 31.2 (10233) |
|  | Poorest | 2.8 (695) | 70.5 (5327) | 18.4 (6022) |
|  |  |  |  |  |
| **Human capital** | University | 22.3 (5615) | 4.7 (351) | 18.2 (5966) |
|  | Secondary | 51.9 (13096) | 33.0 (2493) | 47.6 (15589) |
|  | Primary | 23.6 (5963) | 54.4 (4110) | 30.7 (10073) |
|  | None | 2.2 (557) | 7.9 (598) | 3.5 (1155) |

SD: Standard deviation a. Empty quintile

Figure S1 Histogram with Kernel-density estimates distribution of HWI Colombia DHS 2005 (all households N=38211)

Figure S2 Histogram with Kernel-density estimates distribution Physical capital score

DHS 2005 (all households N=38211)

Figure S3 Histogram with Kernel-density estimates distribution Public capital score

Colombia DHS 2005 (all households N=38211)
